# A Novel Peptide Derived from Human Pancreatitis-Associated Protein Inhibits Inflammation *In Vivo* and *In Vitro* and Blocks NF-Kappa B Signaling Pathway

**DOI:** 10.1371/journal.pone.0029155

**Published:** 2011-12-14

**Authors:** Xiaolu Yang, Huiyi Jin, Kun Liu, Qing Gu, Xun Xu

**Affiliations:** 1 Department of Ophthalmology, Shanghai First People's Hospital, Shanghai Jiaotong University, Shanghai, People's Republic of China; 2 Shanghai Key Laboratory of Fundus Disease, Shanghai, People's Republic of China; Sun Yat-sen University Cancer Center, China

## Abstract

**Background:**

Pancreatitis-associated protein (PAP) is a pancreatic secretory protein belongs to the group VII of C-type lectin family. Emerging evidence suggests that PAP plays a protective effect in inflammatory diseases. In the present study, we newly identified a 16-amino-acid peptide (named PAPep) derived from C-type lectin-like domain (CTLD) of human PAP with potent anti-inflammatory activity using both *in vivo* and *in vitro* assays.

**Methodology/Principal Findings:**

We assessed the anti-inflammatory effect of PAPep on endotoxin-induced uveitis (EIU) in rats and demonstrated that intravitreal pretreatment of PAPep concentration-dependently attenuated clinical manifestation of EIU rats, reduced protein leakage and cell infiltration into the aqueous humor (AqH), suppressed tumor necrosis factor (TNF)-α, interleukin (IL)-6, intercellular adhesion molecule-1 (ICAM-1) and monocyte chemoattractant protein (MCP)-1 production in ocular tissues, and improved histopathologic manifestation of EIU. Furthermore, PAPep suppressed the LPS-induced mRNA expression of TNF-α and IL-6 in RAW 264.7 cells, inhibited protein expression of ICAM-1 in TNF-α-stimulated human umbilical vein endothelial cells (HUVECs) as well as U937 cells adhesion to HUVECs. Western blot analysis in ocular tissues and different cell lines revealed that the possible mechanism for this anti-inflammatory effect of PAPep may depend on its ability to inhibit the activation of NF-kB signaling pathway.

**Conclusions/Significance:**

Our studies provide the first evidence that the sequence of PAPep is within the critically active region for the anti-inflammatory function of PAP and the peptide may be a promising candidate for the management of ocular inflammatory diseases.

## Introduction

Uveitis is a relatively common intraocular inflammatory disease and one of the most damaging ocular conditions that its recurrent nature could lead to cataract, macular edema, glaucoma, and, ultimately, destruction of the intraocular tissues and blindness [1]. Although age-related macular degeneration, glaucoma, and diabetic retinopathy are more prevalent causes of blindness, the relative youth of patients diagnosed with uveitis makes it inevitably one of the ocular diseases with an important socioeconomic impact [2].

Present pharmacological treatment for uveitis primarily includes nonsteroidal anti-inflammatory drugs (NSAIDs), corticosteroids and immunosuppressive agents such as cyclosporin A [3]. However, these drugs do not completely control the disease in many patients and long term application of these drugs may result in multiple adverse effects such as cataract, glaucoma, susceptibility to microbial infection and nephrotoxicity [4]–[7]. Hence, there is a need for therapeutics with safer modes of action. Small peptides are emerging and promising agents in developing new therapeutics for different diseases. The advantages of some peptides as drugs over proteins include sufficient penetration capability, potential efficacy, less toxicity, lower immunogenicity and controllable production, which make them potential alternatives for ocular application. Thus, researchers began to show interests in the active fragments of large proteins that target inflammation [8]–[11].

Pancreatitis-associated protein (PAP) is a 16.6 kDa pancreatic secretory protein belongs to the group VII of C-type lectin family. It is comprised of a short N-terminal domain and a large C-type lectin-like domain (CTLD) that spans the rest of the protein [12]–[15]. Emerging evidence supports the notion that PAP plays a protective effect against inflammatory damage in pancreatic [16], [17] and extrapancreatic [18], [19] inflammatory conditions, and CTLD is the main active region. In a rat model of acute pancreatitis, the infusion of anti-PAP1 antibodies worsened the pancreatic inflammatory response and PAP1 treatment prevented tumor necrosis factor (TNF)-α-induced Nuclear Factor kappa B (NF–κB) activation in macrophages [16]. In addition, PAP prevented fMLP-induced vasoconstriction and edema formation in the isolated rabbit lung and protected the lung from neutrophil-induced injury [18]. Studies by Gironella et al. [19] found that PAP is anti-inflammatory in patients with inflammatory bowel disease. It was also reported that PAP inhibited macrophage activation by down-regulating the synthesis of TNF-α and interleukin (IL)-6, thereby promoting an anti-inflammatory state [20], which are possibly mediated through the inhibition of NF-κB pathway [21]–[23].

In the present study, we chose three conserved sequence segments from CTLD of human PAP to construct three small peptides. We used bioinformatics methods to evaluate their biological activities and investigated these peptides' effects on ocular inflammation, using an established animal model, endotoxin-induced uveitis (EIU), and finally focused on one of these three peptides with the sequence of ASLSRSTAFLRWKDYN (named PAPep). We found that PAPep peptide diminished the inflammatory reaction in EIU rats, suppressed the LPS-induced cytokine release in ocular tissues and macrophage RAW 264.7 cells, and inhibit adhesion molecule expression in stimulated endothelial cells. In order to gain a further mechanistic insight, we assessed whether PAPep modulates activation of NF-κB in ocular tissues and two different cell lines, namely, RAW264.7 cells and HUVECs, as models of the cell types that orchestrate the inflammation present in uveitis. These interesting results suggest that PAPep peptide is within the key active region of PAP CTLD and a potential anti-inflammatory therapeutic agent.

## Materials and Methods

### Ethics Statement

All studies involving animals were conducted in strict accordance with the Association for Research in Vision and Ophthalmology Statement for the Use of Animals in Ophthalmic and Vision Research. The protocol was approved by the Ethics Committee of Shanghai First People's Hospital, Shanghai Jiaotong University, Shanghai, China (Permit Number: 2009–0086). All surgeries were performed under sodium pentobarbital anesthesia, and all efforts were made to minimize suffering.

### Peptide Identification and Synthesis

**Figure 1:**
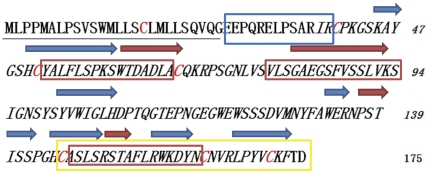
Amino acid sequence of human PAP. The secondary structure is depicted above the primary sequence. Red arrows above the sequences correspond to helical regions. Blue arrows above the sequences correspond to beta-strand regions. Signal sequence is indicated underlined. The blue boxed region corresponds to the N-terminal PAP domain. The C-type lectin domain is indicated in italic. The yellow boxed region is the C-type lectin consensus sequence. Cysteines involved in the formation of the three disulfide bonds are highlighted in red. Three distinct regions from which peptides are processed out are boxed in red as follows: P1, PAP[52–67]; P2, PAP[79–94]; P3 (PAPep), PAP[147–162].

In order to identify the sequence segment that is most probably within the critically active region from CTLD of human PAP, we first did comparative alignment of the amino acid sequence of human PAP with mouse, rat, dog and sheep PAP isoforms using the ClustalW program and chose regions with higher homology amongst various species. Second, we investigated the second structure of PAP and found out that there are several helical and beta-strand regions within the protein sequence (Figure 1). It is reported that helical conformation is a feature of some antibacterial peptides [24]–[27]. So we selected sequence regions with helical conformation. Based on the results of the combined analysis, we ultimately chose three sequence segments derived from the CTLD of human PAP to synthesize three peptides 16 amino acids in length (P1: YALFLSPKSWTDADLA, P2: VLSGAEGSFVSSLVKS, P3: ASLSRSTAFLRWKDYN, P3 named PAPep here). Prior to synthesis, the biological activity of the three peptides were analyzed using bioinformatics methods [28], [29], which include physicochemical parameters, analysis of antigenicity, surface accessibility and mean hydrophilicity.

The three peptides was synthesized and purified by ChinaPeptides Co., Ltd. in Shanghai, PR China with a high-efficiency solid-phase method using an automatic peptide synthesizer (Symphony; Protein Technologies, Tucson, AZ). These purified peptides were characterized by means of amino acid analysis, high-performance liquid chromatography (HPLC, analytical; Shimadzu, Kyoto, Japan) and mass spectrometry (MS, Finnigan TSQ 7000; Thermo, Waltham, MA) with the purity more than 95%. In order to identify whether the PAPep function of anti-inflammation is sequence-dependent, a scrambled peptide (PAPS) was simultaneously synthesized and used as a negative control for the following *in vivo* and *in vitro* experiments. Synthetic peptides were reconstituted in phosphate-buffered saline (PBS; pH7.4) for the experiments.

### Induction of EIU and Drug Treatment Protocol

Six- to eight-week-old Wistar rats (160–180 g) were obtained from Shanghai Laboratory Animal Center, Chinese Academy of Sciences. Animals were maintained in a 12 h light/12 h dark cycle. Food and water were supplied ad libitum. EIU was induced by a single footpad injection of 100 µl sterile pyrogen free saline containing 200 µg lipopolysaccharide (LPS) from *Escherichia coli* (Sigma-Aldrich, Inc., St. Louis, MO). A separate group received the same volume of sterilized saline in one foot pad for comparison with the LPS group (the control group). All animals were anesthetized and pupils were dilated before experimental manipulations. Different concentration peptides (P1–P3 and PAPS) were injected intravitreally to both rat eyes, using a pump microinjection apparatus under a dissecting microscope, an hour before LPS was administered, as previously described [30]. LPS group also received intravitreal injection of PBS (vehicle) in a comparable volume at the same time points as the peptides. Dexamethasone sodium phosphate (10 µg/injection; Shanghai General Motors Pharmaceutical Industry Company Limited, China) was used as a positive control for anti-inflammatory activity on EIU. Twenty-four hours after LPS injection, rats were killed and processed for clinical, biological and immunohistochemical analysis. Five normal rats were injected intravitreally with PAPep (10 µg/eye) to detect any proinflammatory effect in the eye and examined with a slit-lamp biomicroscope for 3 days to determine the short-term safety of the treatment. The experiments were repeated at least three times to ensure reproducibility.

### Clinical Scoring

Animals were examined with a biomicroscope 24 h after LPS injection. Clinical manifestations of EIU were graded from 0 to 4 in a blinded fashion according to the previously reported scoring system [31]: 0 = no inflammatory reaction; 1 = discrete dilation of iris and conjunctival vessels; 2 = moderate dilation of iris and conjunctival vessels with moderate flare in the anterior chamber; 3 = intense iridal hyperemia with intense flare in the anterior chamber; 4 = same clinical signs as 3 with presence of fibrinoid exudation in the pupillary area and miosis. No signs of uveitis were observed in the animals at the beginning of each experiment. Clinical EIU was considered positive when >1. EIU clinical data shown were representative of 3 sets of experiments and presented as mean ± standard deviation (SD).

### Number of Infiltrating Cells and Protein Concentration in Aqueous Humor

Immediately after the biomicroscope examination, the animals were killed with an overdose of anesthesia. Aqueous humor (AqH) was collected immediately from both eyes by an anterior chamber puncture (30–40 µl/rat), using a 30-gauge needle under a surgical microscope. For cell counting, the sample was suspended in an equal amount of Türk stain solution, and the cell number was counted with a hemocytometer under a light microscope. The number of cells per field was manually counted by two independent researchers, and the number of cells per microliter was obtained by averaging the results of four fields from each sample. Total protein concentration was measured by a Coomassie Plus (Bradford) Assay Kit (Pierce, Rockford, IL, USA). The AqH samples were stored in ice water until testing, and cell counts and total protein concentrations were measured on the day of sample collection.

### Enzyme-Linked-Immunosorbent Assay (ELISA)

The AqH from both eyes of a rat were centrifuged at 2500 rpm for 20 minutes at 4°C, to obtain the supernatant. Protein levels of TNF-α, IL-6 in the aqueous humor were determined by the rat TNF-α and IL-6 ELISA kits (R&D Systems, Minneapolis, MN, USA) according to the manufacturer's instruction. The minimum detectable dose of rat TNF-α and IL-6 is about 5.0 pg/ml and 21.0 pg/ml respectively.Soon after collection of aqueous humor, both eyes were immediately enucleated. The iris ciliary body (ICB) and retina complex of each rat was carefully isolated and placed into 200 µl lysis buffer supplemented with protease inhibitors and then sonicated. The lysate was centrifuged at 15,000 rpm for 20 minutes at 4°C. The protein levels of intercellular adhesion molecule-1 (ICAM-1) and monocyte chemoattractant protein (MCP)-1 in the supernatant were determined with the rat ICAM-1 (R&D Systems, Minneapolis, MN, USA) and MCP-1 (Invitrogen, Grand Island, NY) ELISA kits, respectively, according to the manufacturer's protocols. The minimum detectable dose of rat ICAM-1 and MCP-1 is about 2.0 pg/ml and 8.0 pg/ml respectively. All measurements were performed in duplicate. The data represent the mean of eight assay results ± SD.

### Histopathological Studies

A separate set of rats treated in the same way as described earlier were used for the histological study. 24 h after LPS injection, the eyes were enucleated immediately and stored in a mixture of 10% formalin and 2.5% glutaraldehyde for 24 h. After dehydration and paraffin embedment of the eyes, five micrometer sagittal sections near the optic nerve head were obtained. For histopathological evaluation, tissue sections were deparaffinized with xylene and stained using hematoxylin and eosin. Infiltrating inflammatory cells in ICB and posterior vitreous were counted and identified histologically in a masked fashion by an ocular pathologist using a counting grid at 400×magnification. The number of infiltrating inflammatory cells in six sections per eye in the ICB and in three sections per eye in the posterior vitreous was averaged and recorded.

### Cell Culture

Mouse macrophage-like cell line RAW264.7 (ATCC, Manassas, VA) were cultured in Dulbecco's modified Eagle's medium (DMEM; Invitrogen-Gibco, Grand Island, NY) supplemented with 10% fetal bovine serum (FBS; ScienceCell, San Diego, CA), 100 U/ml penicillin, 100 mg/ml streptomycin at 37°C in a humidified atmosphere containing 5% CO_2_. Human umbilical vein endothelial cells (HUVECs) (ScienCell™ no.: 8000) and endothelial cell culture media (ECM) were obtained from ScienCell Research Laboratories (San Diego, CA, USA). U937 human monocyte-like cell line (ATCC, Manassas, VA) were maintained in RPMI-1640 containing 1800 mg/l NaHCO3, 4500 mg/l glucose, and 110 mg/l sodium pyruvate, supplemented with 10% FBS, 100 U/ml penicilin and 100 mg/ml streptomycin at 37°C in a humidified atmosphere with 5% CO_2_.

### Cell Viability Assay

RAW 264.7 cells and HUVECs were grown in a final volume of 100 µl of culture medium per well in 96-well plates (2×10^4^ cells/well) and were serum-starved overnight. To measure cytotoxicity, cells were treated with various concentrations of peptides (1, 10, 50, 100 µM) and incubated for another 24 h. Cell viability was measured at the end of treatment by the addition of 20 µl of the 3-(4,5-dimethylthiazol-2-yl)-5-(3-carboxymethoxyphenol)-2-(4-sulphophenyl)-2H-tetrazolium (MTS) reagent for 3 h at 37°C. The optical density was measured spectrophotometrically at 490 nm on a microtiter plate reader (Bio-Rad, Model 680, Hercules, CA). The experiments were repeated three times. Results are expressed as a percentage of the viability rate, which was calculated as (optical density of drug treated sample/PBS-treated control sample) ×100%.

### Assessment of PAPep Effects on Stimulated TNF-α and IL-6 Expression in RAW264.7 Cells

RAW264.7 cells were grown to confluence in 6-well plates and starved in serum-free DMEM for 12 h before addition of the LPS or peptides. PAPep at various concentrations (1, 10, 50 µM) was added to the culture medium an hour before LPS stimulation (100 ng/ml). PAPS (50 µM) and dexamethasone (10 µM) were also added to the culture medium at the same time points as PAPep. Spontaneous cytokine production was determined using the cell culture medium without LPS. After an additional incubation of 6 h, total RNA was obtained to evaluate changes in TNF-α and IL-6 mRNA expression using quantitative real time reverse transcription-polymerase chain reaction (RT-PCR) analysis on a LightCycler detection system (Roche Applied Science, Basel, Switzerland).

Total RNA was extracted from cultures with TRIzol Reagent (Invitrogen, Grand Island, NY) according to the manufacturer's protocol. cDNA from each sample was synthesized from 2 µg of total RNA using random hexamers and expanded by reverse transcription with a commercially available kit (RevertAidTM First Strand cDNA Synthesis Kit, Fermentas). Quantitative real time PCR was performed with SYBR Premix Ex TaqTM (Takara, TaKaRa Biotechnology, Dalian, China) according to the manufacturer's protocols. The reaction conditions were 40 cycles of two-stage PCR consisting of denaturation at 95°C for 30 sec, annealing at 60°C for 30 sec after an initial denaturation step at 95°C for 5 sec. Expression levels of glyceraldehyde 3-phosphate dehydrogenase (GAPDH) gene were used as an internal control. Primer and probes used to analyze TNF-α, IL-6, and GAPDH expression are summarized as follows: for TNF-α: forward primer 5′-GACGTGGAACTGGCAGAAGAG-3′, reverse primer 5′-TTGGTGGTTTGTGAGTGTGAG-3′; for IL-6: forward primer 5′-TCTTGGGACTGATGCTGGTG-3′, reverse primer 5′-TTGGGAGTGGTATCCTCTGTGAA-3′; for GAPDH: forward primer 5′-TGACCACAGTCCATGCCATC-3′, reverse primer 5′-GACGGACACATTGGGGGTAG-3′. Gene expression was quantitated relative to GAPDH; relative expression of the target gene was calculated as 2^−ΔddCt^, where dCt is the difference between the threshold cycle (Ct) for the gene of interest and the Ct for GAPDH. In each experiment, the value of the relative expression of the control sample (untreated) was given a value of 1 and the expression of other treatments was plotted relative to the control. All samples were analyzed in duplicate and repeated at least three times.

### Immunofluorescence Staining for Detection of NF-κB p65 in RAW264.7 Cells

NF-κB p65 translocation into the nucleus, as an index of NF-κB activation, was measured using Immunofluorescence staining. Cells were plated on to cover slips to 70% confluence and preincubated for an hour with 50 µM PAPep and subsequently incubated with 100 ng/ml LPS for 30 min. Cells were washed in PBS and fixed with 3.7% paraformaldehyde and permeabilized with 0.1% Triton X-100. Slides were blocked with 10% goat serum (Vector Laboratories, Burlingame, CA) for 1 h and incubated overnight at 4°C with anti-NF-κB p65 antibody (1∶1000, Cell Signaling Technology Inc., Beverly, MA), followed the next day by a one-hour incubation at room temperature with anti-rabbit IgG antibody labeled with Alexa Fluor 555 (Molecular Probes, Eugene, Oregon, USA). Sections were mounted with Vectashield mounting medium with DAPI (Vector Laboratories, Burlingame, CA) and visualized and photographed under a confocal laser scanning microscopy (Zeiss LSM510; Carl Zeiss, Thornwood, NY). The number of cells with p65 nuclear translocation in six random fields were counted in a masked fashion and expressed as a percentage of the number of translocated cells in comparison to that of total cells. Experiments were performed in triplicate and repeated at least three times.

### Quantification of PAPep Effects on NF-κB Activation in Ocular Tissue and Different Cell Types

The phosphorylation of NF-kB p65 subunit, particular on serine residues 536 in the C-terminal transactivation domain, plays an important role in regulating the expression of various genes encoding pro-inflammatory cytokines and adhesion molecules. To further prove that PAPep might play a role in the regulation of phosphorylation of NF-kB p65^Ser536^, Western blot analysis was performed in both ocular tissues and different cell types.

The retina and ICB complex obtained 3 h after EIU induction were lysed using the ProteoJET™ Mammalian Cell Lysis Reagent from Fermentas under conditions for preparation of protein extracts. RAW 264.7 cells as model of immune cells, and human umbilical vein endothelial cells (HUVECs) were preincubated for an hour with various doses of PAPep (1 µM, 10 µM and 50 µM) and activated with LPS (100 ng/ml) or TNF-α (10 ng/ml) for 30 minutes. Then, whole cell extracts were obtained. Protein concentration of the homogenate was determined following Bradford's colorimetric method. Each sample containing 50 µg total protein was separated by SDS-PAGE and electroblotted to polyvinylidene fluoride membrane (Millipore, Billerica, MA, USA). After blocking nonspecific binding with 5% BSA, the membranes were incubated with primary antibodies against phosphor-NF-κB p65 (Ser536) or NF-κB p65 (all 1∶1000, Cell Signaling Technology Inc., Beverly, MA) at 4°C overnight. After incubation with horseradish peroxidase-conjugated anti-rabbit antibody (1∶1,000; R&D Systems, Minneapolis, MN, USA) for 2 h at room temperature, proteins were visualized by Immobilon Western Chemiluminescent HRP Substrate (Millipore, Billerica, MA, USA). The relative band intensity for phosphor-NF-κB p65 was calculated in comparison to that for NF-κB p65 after densitometry analysis with computer software, ImagePro Plus 6.0 (Media Cybernetics Inc., Bethesda, MD, USA).

### Monocytic Cell–Endothelial Cell Adhesion Assay

The impact of PAPep on adherence of U937 cells to TNF-α-activated HUVECs was evaluated as described previously [32]–[33]. Briefly, HUVECs at a density of 1.0×10^5^ cells/well were cultured in 10% FBS ECM on a 24-well plate. When reaching 90%–95% of confluence, the cells were starved in serum free ECM medium for 12 h, and then exposed to PAPep (1, 10, 50 µM) or PAPS (50 µM) for 18 h, followed by 6 h stimulation with TNF-α (10 ng/ml). U937 cells freshly harvested were labeled with CM-H_2_DCFDA (10 µM) (Invitrogen, Grand Island, NY) in 10% FBS RPMI 1640 medium at 37°C for 30 min. After extensively washing with PBS, the CM-H2DCFDA -labeled U937 cells were gently added in triplicate into the HUVEC wells that had been treated with PAPep or PAPS, and then incubated at 37°C for another 30 min. Non-adhered U937 cells were then gently washed out with 1% FBS PBS and HUVECs were fixed with 4% paraformaldehyde in PBS for 10 min, followed by 20 min at room temperature with rhodamine phalloidin from Molecular Probes (Eugene, Oregon, USA). Random fields were imaged under a confocal laser scanning microscopy (Zeiss LSM510; Carl Zeiss, Thornwood, NY). The numbers of adhered U937 cells were counted in a masked fashion and expressed as the number of adhered cells per field. Experiments were performed in triplicate and repeated at least three times.

### Effects of PAPep on Adhesion Molecule Expression in Endothelial Cells

HUVECs were incubated for 24 h at 37°C with 5% CO_2_, starved overnight, treated with various concentrations of PAPep (1 µM, 10 µM and 50 µM) an hour before stimulation with 10 ng/ml TNF-α and harvested 6 h later. Whole cell extracts were obtained and primary antibodies against ICAM-1 (1∶1000; Epitomics Inc., Burlingame, CA) were used to detect protein expression of ICAM-1 by Western blot as described above. GAPDH (1∶1000; KangChen Bio-tech, Shanghai, China) was used as loading control.

### Statistical Analysis

Data are expressed as mean ± SD of at least three independent experiments. Severity of EIU was analyzed using the nonparametric Mann-Whitney U test. Differences between mean values of multiple groups were analyzed by one-way analysis of variance with Dunnett's test for post hoc comparisons. A P-value less than 0.05 was considered statistically significant. All computations were performed with the SPSS16.0 (Chicago, IL) software.

## Results

### Identification of PAPep Peptide as a Potential Inflammatory Inhibitor from the C-type lectin domain of PAP

We first analyzed the peptides' characteristics including isoelectric point, half-life, net charge at PH 7.0 using ExPASy (http://www.expasy.ch/tools/protparam.html). As shown in Table 1, these are all small peptides with molecular mass less than 2 kDa, half-life between 2.8–100 h. Unlike the other two peptides, PAPep (P3) is slightly positively charged at neutral pH (charge +2.0) and has an isoelectric point of 9.99, which means that under neutral pH environments the peptide retains its positive charge. This cationic property is believed to be very important for peptide interactions with negatively charged membranes and/or cell walls of Gram-negative bacteria [34], [35].

**Table 1:** The properties of the three newly identified peptides.

Peptide	P1	P2	PAPep(P3)
**Number of Amino Acids**	16	16	16
**Molecular Weight (Da)**	1798	1566.8	1915.1
**Isoelectric Point**	4.21	5.97	9.99
**Estimated Half-life (h)**	2.8	100	4.4
**Net Charge at PH 7.0**	-1	0	2

Using DNAStar software, we then analyzed the mean hydrophilicity (Kyte & Dolittle), antigenic index (Jameson-Wolf) and surface probability (Emini) of these 3 peptides (data not shown). PAPep (P3) has higher mean hydrophilicity, antigenic index and surface probability compared with the other two peptides, suggesting that PAPep possesses better properties of hydrophilicity, antigenicity, and surface accessibility, which are essential for it to perform biological functions *in vivo*.

**Figure 2:**
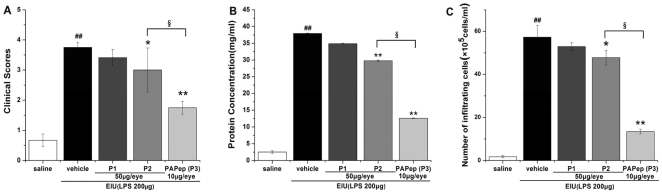
Anti-inflammatory effects of PAP peptides (P1–P3) in EIU rats. The rats were treated with PAP peptides (P1–P3) in different concentrations 1 h before LPS (200 µg) injection, and were evaluated 24 h after LPS challenge. Clinical scores (A) were graded in a blinded fashion. Protein levels (B) and cell count (C) were assessed in the AqH 24 h after EIU. Data are expressed as mean±SD (n = 8 per group). ##, P<0.01 compared with control group; *, P<0.05 or **, P<0.01 compared with vehicle-treated group; §, P<0.01 compared with P2-treated group.

To identify the key active region in CTLD domain of PAP protein in anti-inflammation, we examined the potential effects of the 3 peptides (P1–P3) on ocular inflammation in EIU rats by grading of clinical score, measurement of protein concentration and cell infiltration in the AqH. As shown in Figures 2A–2C, little anti-inflammatory effect was observed with P1 peptide, while P2 and PAPep (P3) can both attenuate the clinical manifestation in EIU rats compared with vehicle-treated group. However, P2 couldn't exhibit its anti-inflammatory effect until reaching a relatively high concentration of 50 µg/eye, and PAPep showed more significant inhibition effect at concentration as low as 10 µg/eye, which was 5 times lower than that of P2 (data of dose responses for P2 are not shown). Based on the results of bioinformatics analysis and *in vivo* experiment, we chose to further study the anti-inflammatory activity of PAPep (P3).

### Clinical Scores, Protein Concentration, and Number of Cells in the Aqueous Humor after Injection of PAPep and LPS

**Figure 3:**
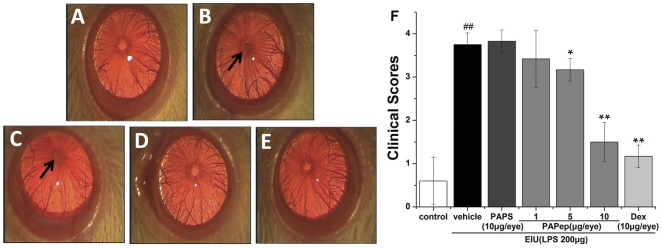
The effects of PAPep on clinical development of EIU. The rats were treated with vehicle (PBS), PAPS (10 µg/eye), PAPep (1, 5, 10 µg/eye) or dexamethasone (10 µg/eye) 1 h before LPS (200 µg) injection, and were evaluated 24 h after LPS challenge with biomicroscope examination. Clinical manifestation of EIU in Wistar rats are shown in Figure A–E. Rats of control group (A) showed no inflammation in the anterior chamber. Severe inflammation was observed in vehicle-treated rats (B) and rats treated with PAPS (C). Note the fibrinous pupillary membrane (arrow). In the group of EIU rats treated with PAPep (10 µg/eye; D), attenuation of inflammation was observed compared with vehicle-treated rats. A similar inhibition was also found for intravitreal pretreatment with dexamethasone (E). (F) Effect of various dosages of PAPep on clinical scores in EIU rats, which were graded in a blinded fashion 24 h after EIU. Data are expressed as mean±SD (n = 8 per group). ##, P<0.01 compared with control group; *, P<0.05 or **, P<0.01 compared with vehicle-treated group. Dex, dexamethasone.

Then, we further explored the effect of intravitreal injection of PAPep on ocular inflammation in the anterior segment and compared its effect with dexamethasone. As shown in Figure 3B, severe inflammation was found in the anterior chamber 24 h after LPS administration, and the clinical score for the EIU rats was 3.75±0.27. By contrast, PAPep treatment reduced the inflammation (Fig. 3D) and improved clinical score in a dose dependent manner (Fig. 3F). By intravitreal injection of 10 µg/eye PAPep, clinical score was significantly (P<0.01) reduced to 1.50±0.45 compared with vehicle-treated group, and the effect was comparable with that of the positive control group receiving dexamethasone (1.17±0.26) (Fig. 3E and 3F). However, intravitreal injection of PAPS had no therapeutic effect on ocular inflammation (mean clinical score: 3.83±0.26).

**Figure 4:**
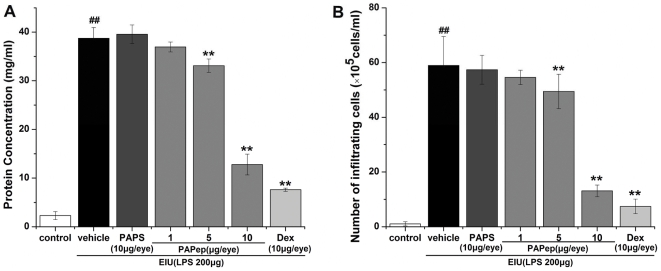
The effects of PAPep on protein leakage and cellular infiltration into the aqueous humor during EIU. The rats were treated with vehicle (PBS), PAPS (10 µg/eye), PAPep (1, 5, 10 µg/eye) or dexamethasone (10 µg/eye) 1 h before LPS (200 µg) injection. Protein levels (A) and cell count (B) were assessed in the AqH 24 h after EIU. Data are expressed as mean±SD (n = 8 per group). ##, P<0.01 compared with control group; **, P<0.01 compared with vehicle-treated group. Dex, dexamethasone.

Consistent with the clinical signs, a very low level of soluble protein (2.31±0.83 mg/ml) and no inflammatory cells were found in the AqH of the control rats. In LPS group, however, the protein levels and number of inflammatory cells in the AqH were 38.74±2.21 mg/ml and 59±11×10^5^ cells/ml, respectively, significantly higher than those in the control group (Fig. 4A and 4B). Relative to vehicle-treated rats, the LPS-induced elevation of soluble proteins and increase number of inflammatory cells were dose-dependently and significantly reduced in animals pretreated intravitreally with PAPep. The calculated reductions over protein leakage and cellular influx caused by PAPep (10 µg/eye) pretreatment were 67 and 78%, respectively. Pretreatment with dexamethasone (10 µg/eye) also significantly reduced the protein leakage and cellular infiltration into the AqH compared with vehicle-treated animals (inhibitions of 80 and 87%, respectively). However, intravitreal pretreatment with PAPS (10 µg/eye) did not reduce the cellular infiltration (57±5×10^5^ cells/ml) and protein leakage (39.57±1.92 mg/ml) into the AqH.

### PAPep Suppressed Histopathological Changes of EIU

**Figure 5:**
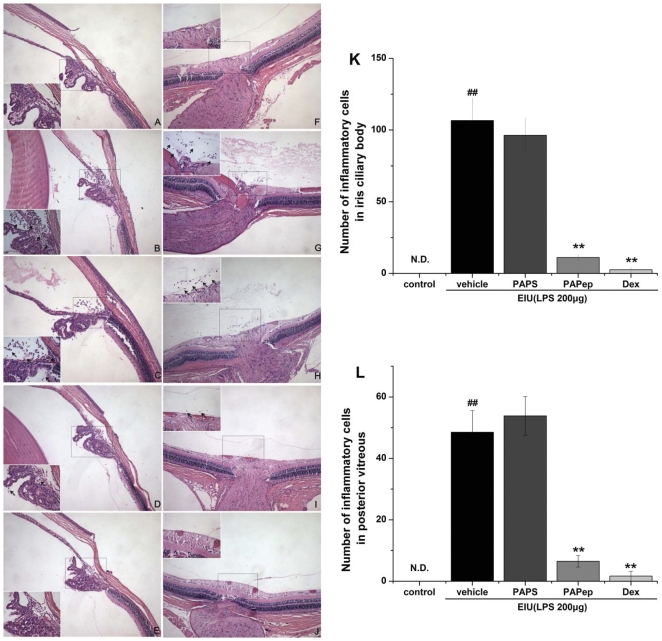
Histological evaluation of EIU rats treated with PAPep. The rats were treated with vehicle (PBS), PAPS (10 µg/eye), PAPep (10 µg/eye) or dexamethasone (10 µg/eye) 1 h before LPS (200 µg) injection. Rats' eyes were enucleated 24 h after LPS stimulation, fixed, sectioned, and stained with H&E. Photographs on the left side show ICB region and those on the right side show posterior vitreous and retina in rat. Rats of control group (A, F) showed no infiltrating cells in the eye. Severe inflammatory cell infiltration was observed in vehicle-treated rats (B, G) and rats treated with PAPS (10 µg/eye; C, H). In the group of EIU rats treated with PAPep (10 µg/eye; D, I), milder uveitis and reduction of cell infiltration were observed compared with EIU rats. A similar inhibition was also found for intravitreal pretreatment with dexamethasone (10 µg/eye; E, J). Arrows: inflammatory cells. Original magnification (A-J)×100; (insets: A-J)×400. (K-L) Number of inflammatory cells infiltrating the eye during EIU. Inflammatory cells in the ICB (K) and posterior vitreous (L) were counted 24 hours after EIU induction. Results are representative of those in six pairs of eyes. Data are expressed as mean±SD (n = 5 per group). ##, P<0.01 compared with control group; **, P<0.01 compared with vehicle-treated group. Dex, dexamethasone; N.D., Not detected.

Histopathological changes of EIU were evaluated and inflammatory cells infiltrating the eye were counted 24 hours after EIU induction. Histological analysis revealed no infiltrating cells in the eyes of animals that had received a footpad injection of sterile saline (control group, Fig. 5A). On the other hand, the histological evaluation from the LPS-treated rats revealed signs of severe uveitis with massive neutrophil and monocyte infiltration, mainly into the ICB (107±16 cells/section; Fig. 5B) but also into the posterior vitreous (49±7 cells/section; Fig. 5G). Pretreatment with PAPep (10 µg/eye) exhibited a significantly milder uveitis and reduced number of inflammatory cells compared with vehicle-treated animals (Fig. 5D and 5I). On average, 11±2 and 7±2 inflammatory cells per ocular section were detected in the ICB and posterior vitreous during EIU, respectively (Fig. 5K and 5I). A similar inhibition was also found for intravitreal pretreatment with dexamethasone (10 µg/eye). In PAPS treatment group, 96±12 and 54±6 inflammatory cells per ocular section were detected in the ICB and posterior vitreous, which was not significantly different from that of the vehicle-treated animals. In addition, there was no sign of cytotoxicity in any of the treatment groups. Eye structures exhibited no histologic alteration other than some preparation artifacts.

### PAPep Decreased LPS-induced TNF-α and IL-6 Expression in AqH during EIU

**Figure 6:**
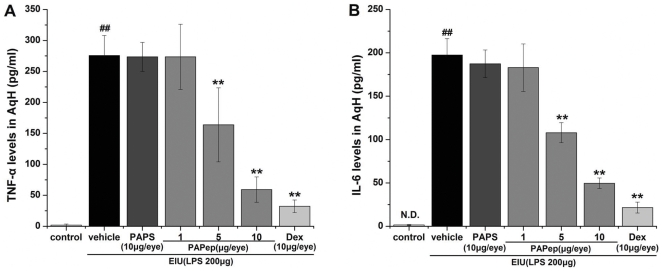
Effects of PAPep on cytokine production in the AqH during EIU. Vehicle (PBS), PAPS (10 µg/eye), PAPep (1, 5, 10 µg/eye) or dexamethasone (10 µg/eye) was applied intravetreally to both rat eyes 1 h before LPS (200 µg) administration. TNF-α (A) and IL-6 (B) levels were assessed in the AqH 24 h after EIU. Data are expressed as mean±SD (n = 8 per group). ##, P<0.01 compared with control group; **, P<0.01 compared with vehicle-treated group. Dex, dexamethasone; N.D., Not detected.

Cytokines such as TNF-α and IL-6 are important molecules in inflammatory processes and induced by various factors, including bacterial endotoxin LPS [36]. We investigated whether PAPep suppressed LPS-induced TNF-α and IL-6 release in EIU rats. In saline-treated animals (control group), undetectable or very low levels of TNF-α and IL-6 were found in the AqH. As expected, up-regulated levels of TNF-α and IL-6 were present in rat AqH 24 h after LPS stimulation. When administered intravitreally, PAPep (5 µg/eye and 10 µg/eye) significantly reduced the augmentation of both inflammatory cytokines in response to LPS (Fig. 6A and 6B). Especially with 10 µg/eye of PAPep, the inhibitions obtained in the levels of TNF-α and IL-6 were 79 and 75%, respectively. A similar effect was verified in the group pretreated intravitreally with dexamethasone (inhibitions of 88 and 89% for TNF-α and IL-6, respectively). Meanwhile, no effect was verified in the group pretreated intravitreally with 10 µg/eye of PAPS.

### PAPep Reduced Retinal and ICB Expression of ICAM-1 and MCP-1 during EIU

**Figure 7:**
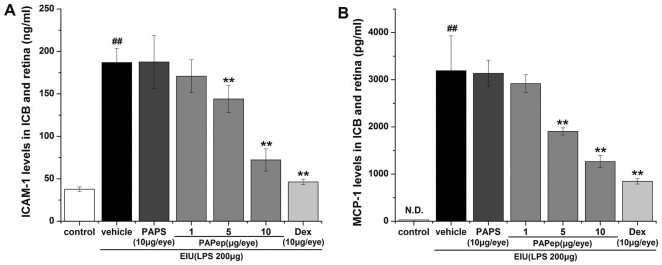
Effects of PAPep on ICAM-1 and MCP-1 expression in the ICB and retina complex during EIU. Vehicle (PBS), PAPS (10 µg/eye), PAPep (1, 5, 10 µg/eye) or dexamethasone (10 µg/eye) was applied intravetreally to both eyes 1 h before LPS (200 µg) administration. Protein levels of ICAM-1 (A) and MCP-1 (B) were assessed in the retina and ICB complex 24 h after EIU. Data are expressed as mean±SD (n = 8 per group). ##, P<0.01 compared with control group; **, P<0.01 compared with vehicle-treated group. Dex, dexamethasone; N.D., Not detected.

To investigate molecular mechanisms underlying retinal leukocyte recruitment and adhesion, protein levels of ICAM-1 (Figs. 7A) and MCP-1 (Figs. 7B) in the retina and ICB complex were evaluated by ELISA. Retinal ICAM-1 and MCP-1 protein levels were significantly (P<0.01 for both) higher in vehicle-treated EIU rats than in saline-treated controls. Intravitral injection of PAPep (10 µg/eye) significantly (P<0.01 for both) reduced ICAM-1 and MCP-1 protein levels by 61 and 60%. Intravitreal treatment with dexamethasone (10 µg/eye) also significantly reduced the expression of ICAM-1 and MCP-1 during EIU. No inhibition effect was found for intravitreal pretreatment with PAPS (10 µg/eye).

### Toxicity Evaluation of Intravitreal Administration of PAPep

Five normal rats received intravitreal injection of PAPep(10 µg/eye) were used to detect proinflammatory effect of the peptide and determine the short-term safety of the treatment. No inflammatory sign in the anterior chamber was detected in any eye in normal rats receiving intravitreal injection of PAPep. Cataract and vitreous opacity was not observed during the observation period (3 days). Histopathologic analysis did not show any evidence of drug-released toxic effect in the PAPep injection group.

### PAPep Inhibited the mRNA Levels of TNF-α and IL-6 and the Translocation of NF-κB in RAW264.7 Cells

**Figure 8:**
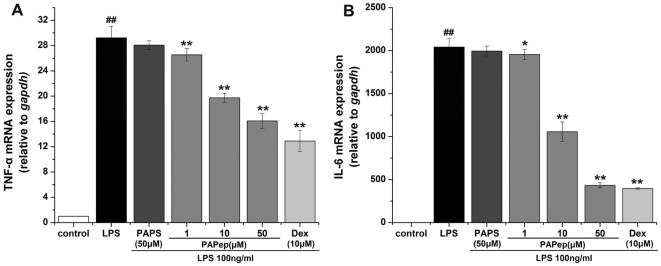
PAPep inhibited LPS-induced TNF-α and IL-6 mRNA expression in RAW264.7 cells. RAW264.7 cells were pretreated with PAPep (1, 10, 50 µM), PAPS (50 µM), or dexamethasone (10 µM) for 1 h, and then stimulated with LPS (100 ng/ml) for 6 h. TNF-α (A) and IL-6 (B) mRNA induction levels were quantified by real-time PCR and normalized to GAPDH mRNA expression. All samples were analyzed in duplicate and repeated at least three times. Data are expressed as mean±SD. ##, P<0.01 compared with control group; *, P<0.05 or **, P<0.01 compared with LPS group. Dex, dexamethasone.

**Figure 9:**
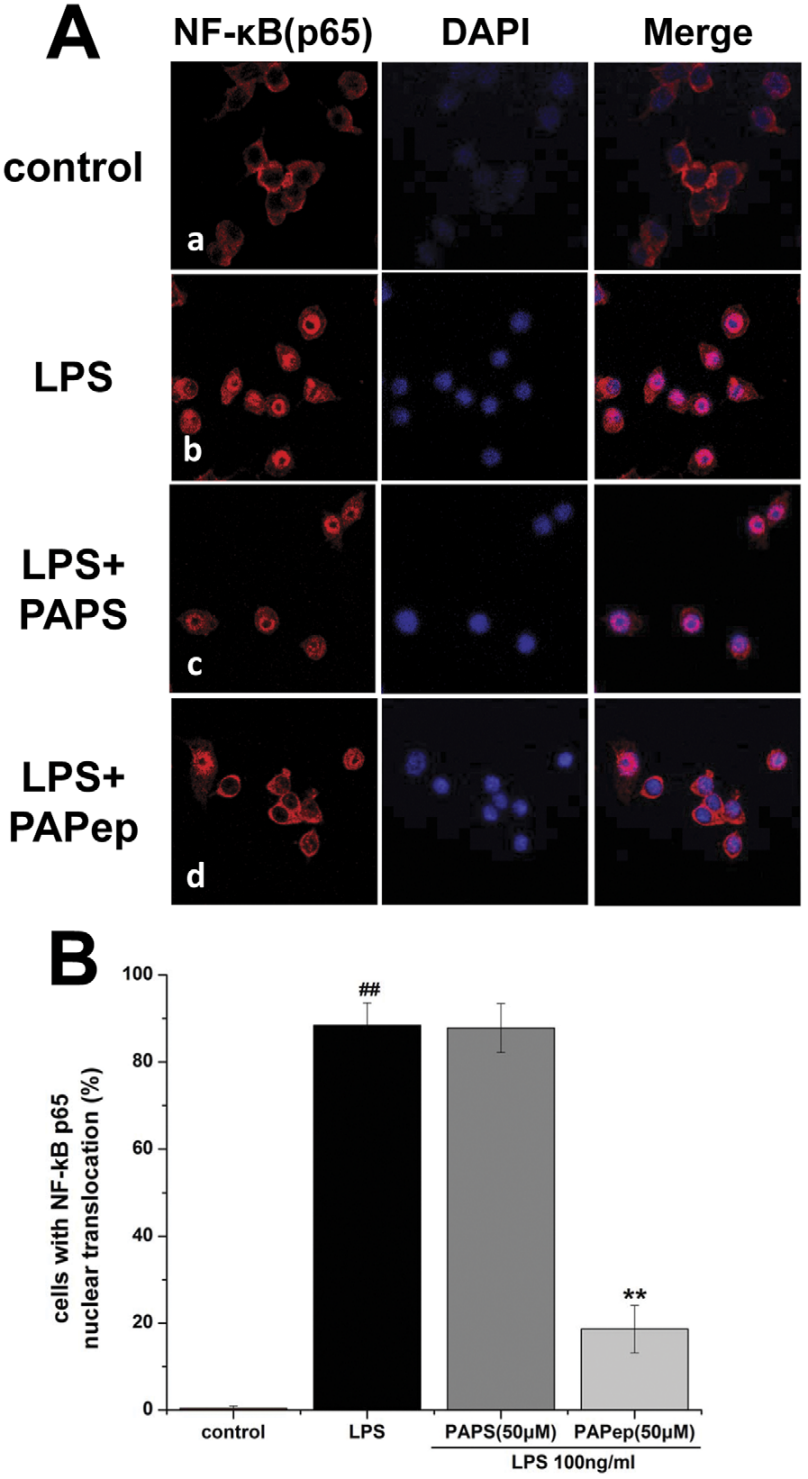
Effect of PAPep on nuclear translocation of NF-κB in RAW264.7 cells. (A) The intracellular location of NF-κB p65 was determined in RAW264.7 cells by immunofluorescence using an anti-NF-κB p65 antibody with Alexa Fluor 555 labelling (red fluorescence), and the nuclei were counterstained with DAPI (blue fluorescence). (a) Untreated cells exhibit the localization of NF-κB in the cytoplasm. Cells stimulated with 100 ng/ml LPS (b) and 50 µM PAPS(c) display a significant increase in the translocation of NF-κB into the nucleus. (d) Stimulated cells in the presence of 50 µM PAPep maintained predominantly cytoplasmic NF-κB immunostaining, indicating inhibition of NF-κB translocation. Results are displayed individually or as merged images (confocal fluorescence microscopy). Data represent one of three experiments with similar results. Scale bars, 10 µm. (B) Nuclear NF-κB was quantitated by visual fluorescent microscopy. The number of cells with p65 nuclear translocation in six random fields were counted in a masked fashion and expressed as a percentage of the number of translocated cells in comparison to that of total cells. ##, P<0.01 compared with control group, **, P<0.01 compared with LPS group.

To further confirm these observations in EIU rats, we investigated the effects of PAPep on the activation of macrophage-like cells, which plays a key role in the orchestration of the inflammatory response during acute uveitis. Activation in vitro of RAW264.7 cells by LPS (100 ng/ml) was monitored by measuring the expression of TNF-α and IL-6 mRNAs, GAPDH mRNA being used as control. Resting RAW264.7 cells showed a low constitutive transcription of pro-inflammatory cytokines, while LPS caused a significant increase in mRNA levels of TNF-α and IL-6 in RAW264.7 cells after 6 h of incubation. Adding PAPep to the culture medium inhibited that induction in a dose-dependent manner, the minimum concentration having a significant effect being 1 µM (Fig. 8A and 8B). However, no inhibitory effect was verified in cells pretreated with PAPS (50 µM). Dexamethasone (10 µM) also significantly inhibited LPS-induced mRNA expressions of TNF-α and IL-6. Importantly, PAPep (50 µM) and Dex (10 µM) had a similar inhibitory effect on LPS-induced mRNA levels of IL-6 (P>0.05). These results suggest that the PAPep prevented TNF-α and IL-6 production through suppressing their gene expressions in LPS-stimulated RAW264.7 cells. Moreover, in an immunofluorescence method, we found that 100 ng/ml LPS caused the nuclear translocation of the p65 subunit of NF-κB, and pretreatment with 50 µM PAPep significantly inhibited the LPS-evoked NF-κB nuclear translocation (Fig. 9). These results suggest that RAW264.7 cells' activation is mediated by the translocation of NF-κB to the nucleus and PAPep exerts anti-inflammatory effects probably through the inhibition of NF-κB translocation.

### PAPep Inhibited Phosphorylation of NF-κB p65 in Ocular Tissues and Different Cell Types

**Figure 10:**
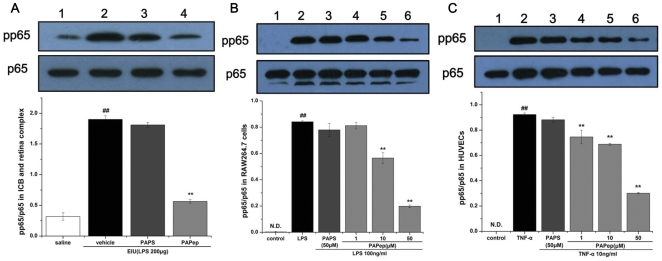
Western blot analysis of protein levels of NF-κB p65. (A) The total and phosphorylation levels of NF-κB p65 was analyzed by Western blot in ICB and retina complex of EIU rats for indicated periods. Lane 1: control; lane 2: LPS and vehicle; lane 3: LPS and PAPS (10 µg/eye); lane 4: LPS and PAPep (10 µg/eye). (B) RAW264.7 cells were pretreated with PAPep (1, 10, 50 µM) or PAPS (50 µM) for 1 h, then stimulated with LPS (100 ng/ml) for 30 min. Total and phosphorylation levels of NF-κB p65 was analyzed by Western blot. Lane 1: control; lane 2: LPS; lane 3: LPS and 50 µM PAPS; lane 4: LPS and 1 µM PAPep; lane 5: LPS and 10 µM PAPep; lane 6: LPS and 50 µM PAPep. (C) HUVEC were incubated with PAPep (1, 10, 50 µM) or PAPS (50 µM) for 1 h, then stimulated with TNF-α (10 ng/ml) for 30 min. Total and phosphorylation levels of NF-κB p65 was analyzed by Western blot. Lane 1: control; lane 2: TNF-α; lane 3: TNF-α and 50 µM PAPS; lane 4: TNF-α and 1 µM PAPep; lane 5: TNF-α and 10 µM PAPep; lane 6: TNF-α and 50 µM PAPep. ##, P<0.01 compared with control group, **, P<0.01 compared with LPS or TNF-α group. Data are expressed as mean±SD of three independent experiments, each performed in duplicates.

The presence of activated NF-κB in the nucleus was further confirmed by Western blot. We measured the phosphorylated p65 NF-κB protein level both in ocular tissues and different cell lines, RAW264.7 cells and HUVECs. Under homeostatic conditions, low levels of phosphorylated p65 NF-κB was found in the ICB and retina complex. EIU, as expected, induced marked phosphorylation of p65 NF-κB in the cells of ICB and retina. Intravitreal PAPep pretreatment significantly reduced the activation of p65 NF-κB in the ICB and retina complex compared with the vehicle-treated group (Fig. 10A). The inhibition in the p65 NF-κB phosphorylation by PAPep (10 µg/eye) was 70.3%, while total p65 NF-κB remained unchanged.

Likewise, no expression of the phosphorated p65 NF-κB protein was detected in unstimulated RAW264.7 cells and HUVECs, but there was strong expression in LPS-stimulated RAW264.7 cells and TNF-α-stimulated HUVECs (Fig. 10B and 10C). Expression of phophorated p65 NF-κB protein decreased in the 10 to 50 µM PAPep group in a dose-dependent fashion in both RAW264.7 cells and HUVECs. These results indicated the potential role of NF-κB in the suppression of inflammatory mediators and pro-inflammatory cytokines by PAPep.

### PAPep Inhibited U937 Cells Adhesion to TNF-α-stimulated HUVECs

**Figure 11:**
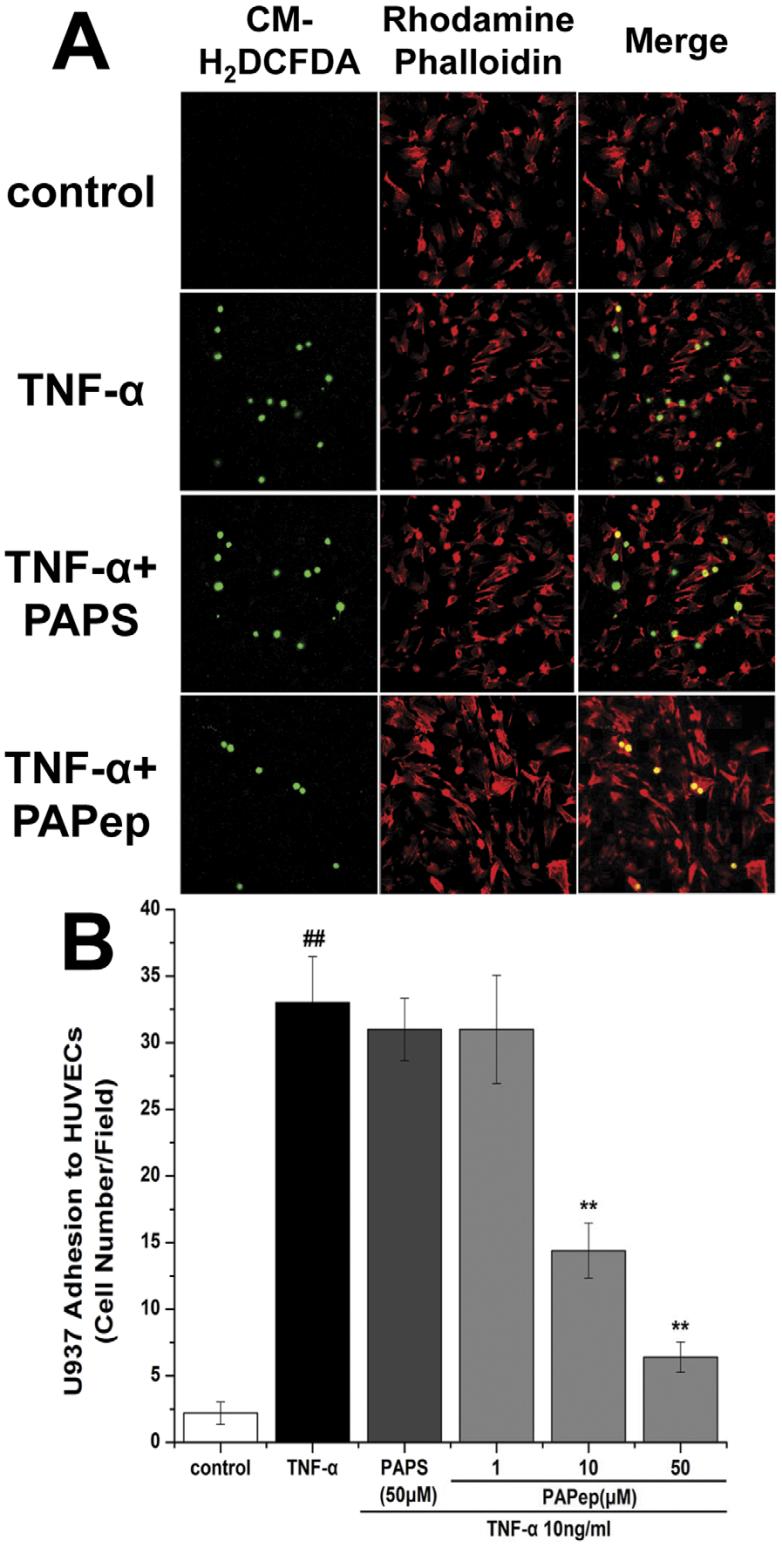
PAPep inhibited U937 cells adhesion to TNF-α-activated HUVEC. (A) HUVEC were incubated with PAPep(1, 10, 50 µM) or PAPS(50 µM) for 18 h, then stimulated with TNF-α (10 ng/ml) for 6 h. CM-H_2_DCFDA-labeled (green fluorescence) U937 cells were seed onto HUVEC and co-cultured for 30 min. After removing the non-adherent cells, adherent cells were fixed and stained with rhodamine phalloidin (red fluorescence), and were counted under a confocal laser scanning microscopy with magnification of 200×. Results are displayed individually or as merged images. Data represent one of three experiments with similar results. Scale bars, 10 µm. (B) Quantitative analysis of the binding of U937 cells to HUVEC presented by bar graphs. ##, P<0.01 compared with unstimulated cells, **, P<0.01 compared with TNF-α-stimulated cells. Data are expressed as mean±SD of results from three independent experiments, each performed in duplicate.

Macrophages are not the only cells that can generate proinflammatory factors during the early stages of uveitis. Endothelial cells also release chemokines such as ICAM-1 through NF-κB activation [33], [37]. We therefore investigated the effect of PAPep on the adhesion of U937 cells to TNF-α-activated endothelial cells, a critical step in vascular inflammation. As shown in Figure 11A, control-confluent HUVECs showed minimal binding of U937 cells, while stimulation of HUVECs with TNF-α for 6 h resulted in a 15-fold increase in firmly adhering U937 cells as compared with resting HUVECs. However, the adhesion of U937 cells to TNF-α-stimulated HUVECs was significantly attenuated by PAPep in a dose dependent manner (Fig. 11E). PAPS didn't show any effect on U937 cells adhesion to HUVECs (Fig. 11C).

### U937 Cells Adhesion to HUVECs Is ICAM-1-dependent

**Figure 12:**
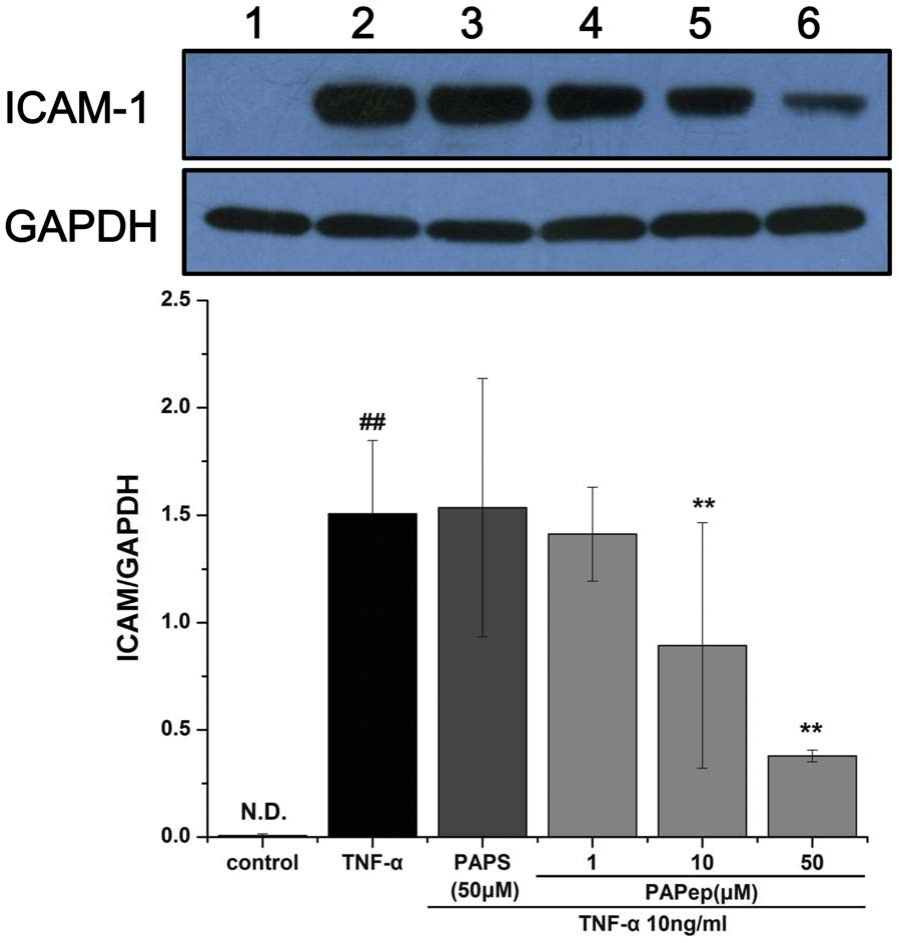
Western blot analysis demonstrating the expression of ICAM-1 protein in HUVECs. HUVEC were incubated with PAPep (1, 10, 50 µM) or PAPS (50 µM) for 1 h, then stimulated with TNF-α (10 ng/ml) for another 6 h. Protein levels of ICAM-1 was analyzed by Western blot. GAPDH was used as loading control. Lane 1: control; lane 2: TNF-α; lane 3: TNF-α and 50 µM PAPS; lane 4: TNF-α and 1 µM PAPep; lane 5: TNF-α and 10 µM PAPep, and lane 6: TNF-α and 50 µM PAPep. ##, P<0.01 compared with unstimulated cells, **, P<0.01 compared with TNF-α-stimulated cells. Data are expressed as mean±SD of results from three independent experiments, each performed in duplicate.

As the expression of adhesion molecules on endothelial cells is a prerequisite for adhesion of leukocytes and monocytes, we investigated the effect of PAPep on TNF-α-induced ICAM-1 expression in HUVECs. Western blot analysis of cell lysates showed that levels of ICAM-1 were undetectable in unstimulated HUVECs, but were significantly increased by TNF-α treatment. PAPep significantly attenuated TNF-α-induced ICAM-1 upregulation in a dose-dependent manner at concentrations ranging from 10 to 50 µM (Figure 12). However, no inhibitory effect was observed by pretreatment with PAPS. Since PAPep similarly interfered with TNF-α-induced up-regulation of NF-κB expression by HUVECs, we supposed that PAPep suppresses U937 cells adhesion to HUVECs via NF-κB signaling.

### PAPEP Is Non-toxic to RAW 264.7 Cells and HUVECs

The results in the cytotoxicity MTS study showed that after 24 h incubation with PAPep, there was no variation in cell viability at concentrations tested (1–100 µM, data not shown). PAPep at 100 µM slightly inhibited RAW264.7 cell proliferation, but no significant differences were observed. No effects on cell viability were observed in cells treated with PAPS (50 µM).

## Discussion

PAP is a 175-amino-acid-long polypeptide containing a large C-type lectin carbohydrate recognition domain linked to a short N-terminal domain. Although there are increasing number of studies showing that the CTLD are involved in regulatory processes pertaining to various aspects of the immune system, such as inflammation [12]–[23], immunity to tumor [38], [39] and virally infected cells [40], it is unclear which fragment of CTLD is the key active region and the underlying mechanism of its anti-inflammation. In this study, we first preliminarily investigate the whole domain by sequence alignment, second structure analysis and bioinformatics analysis to theoretically screen out the most promising regions that might within the key active region of human PAP. Then we examined the potential effects of the selected three peptides on ocular inflammation in EIU rats to ultimately identify which peptide is most likely to possess anti-inflammatory effect. Finally, we showed for the first time a 16-amino acid peptide (PAPep) derived from CTLD of PAP with potent anti-inflammatory activity using both *in vivo* and *in vitro* assays. We demonstrated that intravitreal injection of PAPep effectively attenuates the clinical manifestation in EIU rats, diminishes the cellular infiltration, protein leakage into the anterior chamber, reduces pro-inflammatory cytokines and molecules production, and improves histopathologic manifestation of EIU, while other two synthesized peptides and scrambled peptide had no such effects. Furthermore, PAPep suppresses the LPS-induced mRNA expression of TNF-α and IL-6 in RAW 264.7 cells, and inhibits adhesion molecule expression in stimulated endothelial cells, and these anti-inflammatory effects are probably associated with the inhibition of NF-κB activation. Hence, the amino acid sequence of PAPep studied might be a part of the critically active sequence of PAP CTLD in anti-inflammation and its function is sequence-dependent.

EIU is an experimental model for acute ocular inflammation, produced by footpad injection of endotoxin, the lipopolysaccharide (LPS) component of gram-negative bacterial cell wall [41], [42]. In the ocular tissue, exposure to LPS is known to induce the breakdown of the blood–aqueous barrier, which leads to protein transudate in the anterior chamber, infiltration of macrophages and neutrophils into the eye, and production of multiple proinflammatory mediators, including cytokines and chemokines, such as TNF-α, IL-6, MCP-1 and ICAM-1 [36], [41], [43], [44]. The overproduction of these cytokines is mediated through NF-κB [45], [46]. These clinical, biochemical, immunological and histological characteristics make EIU a suitable in vivo model to evaluate the therapeutic efficacy of drugs for the treatment of inflammatory ocular diseases in humans.

The first report suggesting an anti-inflammatory effect of PAP, the protein that peptide was derived from, was based on an *ex vivo* model of isolated lung. In that model, PAP administration reduced the edema and synthesis of thromboxane A2 induced by fMLP [18]. There are also researchers proving that PAP could block inflammatory responses induced by TNF-α in different cell lines [19], [20], [22]). In the present study, we showed for the first time that PAPep, which was derived from PAP protein, exerted anti-inflammatory effects in ocular inflammation induced by LPS, a TLR4 agonist. We demonstrated that intravitreal injection of PAPep could significantly reduce EIU clinical scores, decrease the number of infiltrating inflammatory cells and the protein leakage into the anterior chamber, and suppress histopathologic changes of EIU. Moreover, the intravetreal administration of PAPep also significantly diminished TNF-α and IL-6 production in the AqH of the eye during EIU, and suppressed the LPS-induced mRNA expression of TNF-α and IL-6 in RAW 264.7 cell as well. These results are in accordance with previous work by Vasseur et al.[20] who reported that in rat macrophages in vitro, PAP could almost completely prevent the induction of TNF-α and IL-6 mRNA expression. However, whether the peptide has anti-inflammatory effect on other TLR agonists remains unknown and need further studies.

It has been reported that leukocytes are markedly attracted to inflamed ocular tissues such as the iris [47], vitreous cavity [48], [49], and retina [50] in the EIU model, with neutrophils and macrophages being major leukocyte constituents. MCP-1 is known as one of the important determinants of leucocyte recruitment, and is up-regulated during EIU [44], [51], [52]. Our study demonstrated that PAPep could directly affect the expression of MCP-1 in ocular tissues in a dose-dependent manner, thus counteracting the inflammatory response by inhibiting leukocyte recruitment into the eye. These results were consistent with what we showed in histopathology study that LPS-induced acute inflammation caused the increase of leukocyte recruitment into the eye, while PAPep treatment significantly reduced the infiltrated leukocytes into the ICB and posterior vitreous cavity.

Increased expression of adhesion molecules is another important factor involved in the pathogenesis of EIU [48], [51], and ICAM-1 is known to be a key molecule in which its reduction results in attenuation of leukocyte adhesion and/or transmigration [49], [53], [54]. Previous reports demonstrated that PAP significantly inhibited TNF-α induced adhesion molecule upregulation in HUVECs in a dose dependent manner [19]. In our study, we found that PAPep significantly decreased the protein levels of ICAM-1 in the inflamed ICB and retina complexes. Moreover, it effectively blocked ICAM-1 expression in TNF-α–stimulated HUVECs by Western blot and inhibited the binding of U937 cells to TNF-α–stimulated HUVECs. These data suggested that PAPep is involved in the regulation of adhesion molecule expression and counteracts the inflammatory response by inhibiting leucocyte recruitment and adhesion in the ocular tissue as well as vascular endothelium, which was agreed with the previous studies.

NF-kB is one of the most ubiquitous transcription factors that regulate gene expressions involved in cellular proliferation, inflammatory responses, and cell adhesion [55], [56]. Upon stimulation with a wide variety of stimuli including LPS and TNF-α, NF-kB translocates from the cytoplasm to the nucleus and plays important roles in sustained inflammatory responses. Li et al. [57] demonstrated a significant upregulation of activated NF-kB in the iris-ciliary body (ICB) during EIU. Previous studies have reported that the anti-inflammatory effects of PAP are mediated through the inhibition of the NF-κB pathway [20]–[23]. Our data also indicate that intravetreal PAPep treatment prevents LPS-induced NF-κB translocation/activation in the ICB and retina tissue in EIU rats and in LPS-stimulated RAW264.7 cells as well. In addition, PAPep also inhibited the TNF-α-evoked activation of NF-κB in HUVECs. These results indicate that PAPep exerted anti-inflammatory effect probably associated with the inhibition of NF-κB activation, and resulted in reduction of ocular inflammatory responses.

In order to observed its safety during injection into the vitreous cavity. The intraocular tolerance of PAPep was tested in normal rats. No inflammation or adverse reaction such as cataract or vitreous opacity was observed in the injected eyes during evaluation by slit-lamp biomicroscopy, suggesting that PAPep is well tolerated in the eye in the short term. We thus preliminarily confirmed the short-term safety of PAPep when administrated intravitreally. However, long-term side effects of the peptide need to be evaluated by multiple injections of the peptide with longer observation period. Further specific toxicological studies should also be performed before any clinical application.

Endotoxin-induced uveitis develops within 24 h of endotoxin challenge, with spontaneous regression within 48 h [41]. Accordingly, we observed the efficacy of PAPep only up to 24 hours after injection, which is relatively short time frame of observation. Hence, long-term study is necessary to determine treatment efficacy of PAPep in chronic uveitis in the future.

A key finding in this study is that PAPep is a newly discovered peptide derived from human PAP. Although its anti-inflammatory activity is not as strong as that of corticosteroids, it has significant merits compared to conventional proteins and large synthetic molecules as discussed above, which make it good candidate for ocular application. However, there are still disadvantages of small peptides as drugs, one of them being short half-life in vivo. Since the predicted half-life of PAPep is only 4.4 hrs, frequent and repeated dosing would be inevitably required for treatment. Therefore, future studies should focus on overcoming these disadvantages by chemical modification or conjugation to a macromolecule to increase its half-life [58]–[59]. Measures could also be taken to improve its delivery system with safer and non-invasive route, such as intravitreous drug delivery system in a biodegradable polymer [60]–[61], to maintain a constant concentration of peptide in the vitreous cavity for an extended period of time to avoid repeated intravitreal injections. Moreover, topical treatment such as eye drops or ointment is another option under the condition that the peptide has the capability of penetrating cornea and anterior chamber, which will also be evaluated in future studies. These efforts might help to promote the development of novel peptide drugs and their clinical applications.

In conclusion, our results suggest that intravitreal injection of PAPep, a newly identified peptide derived from PAP, can significantly attenuate ocular inflammation in EIU rats and dampen the inflammatory damage by affecting diverse components of the inflammatory response, including cytokine production and adhesion molecule expression. The possible mechanism for this effect of PAPep may depend on its ability to inhibit the activation of NF-kB. The exact mechanism through which PAPep exerts its anti-inflammatory effect is under active investigation.
